# Low Birth Weight and Associated Factors in Sudan: A Systematic Review and Meta-Analysis

**DOI:** 10.3390/children13020274

**Published:** 2026-02-16

**Authors:** Elfatih M. Malik, Abdullah Al-Nafeesah, Ashwaq AlEed, Ishag Adam

**Affiliations:** 1 Faculty of Medicine, University of Khartoum, Khartoum P.O. Box 321, Sudan; 2 Department of Pediatrics, College of Medicine, Qassim University, Buraydah 52571, Saudi Arabia; 3 Department of Obstetrics and Gynecology, College of Medicine, Qassim University, Buraidah 52571, Saudi Arabia

**Keywords:** age, low birth weight, anemia, systematic review, meta-analysis, Sudan

## Abstract

**Highlights:**

**What are the main findings?**

- One in every eight newborns had low birth weight.
- Women who were experiencing their first pregnancy, had undernutrition, and did not use folic acid were at higher risk of having LBW newborns.

**What are the implications of the main findings?**

- Greater effort is needed regarding maternal nutrition in order to prevent LBW.
- Women should be encouraged to take folic acid supplements.

**Abstract:**

Background: Low birth weight (LBW) is one of the most significant health issues worldwide, especially in countries with fewer resources. No systematic reviews or meta-analyses on LBW have been conducted in Sudan, the third largest African country. Methods: A systematic review and a meta-analysis were conducted to assess the pooled prevalence of LBW in Sudan and the associated factors. PubMed, Google Scholar, and ScienceDirect were searched for studies on LBW in Sudan. The meta-analysis was performed by calculating the pooled prevalence of LBW. The meta package in R was used for statistical analysis. Results: The final sample comprised 10 studies, with 10,043 neonates enrolled. The overall pooled prevalence of LBW was 13.0% (95% CI [13.0, 14.0]; I^2^ = 47.0%, *p* = 0.05), without significant heterogeneity. Primiparity (adjusted odds ratio [AOR] = 1.4, 95% CI [1.1, 2.1]), a short interpregnancy interval, lack of iron–folic acid supplementation (AOR = 3.33, CI [1.47, 5.88]), a low level of antenatal/perinatal care (AOR = 2.10, 95% CI [1.30, 3.57]), maternal undernutrition (AOR = 1.66, 95% CI [1.09, 2.53]), and decreasing gestational age of pregnancy (AOR = 0.80, 95% CI [0.66, 0.96]) were associated with LBW in different studies. In three studies, anemia was associated with LBW. Conclusions: This review reveals a high prevalence of LBW in Sudan. The factors identified in this review may help health planners and policymakers design and implement preventive interventions for LBW.

## 1. Introduction

The World Health Organization (WHO) defines a low birthweight (LBW) as a weight of less than 2500 g at birth, irrespective of gestational age [1]. There were 20.5 million cases of LBW worldwide—most of which were in Africa—and this number is expected to increase [2,3]. LBW is one of the most significant health issues. It can lead to several perinatal, neonatal, childhood, and adulthood complications, such as intrauterine and intrapartum death [4], neonatal infections [5], neonatal distress syndrome and birth asphyxia [6], increased rates of admission to the neonatal intensive care unit [7], childhood undernutrition [8], neonatal mortality [9], childhood allergies/asthma [10], systematic/arterial hypertension, decreased glomerular filtration rates, chronic kidney disease in childhood and adulthood [11], impaired motor and cognitive function [12], type 2 diabetes mellitus, insulin resistance [13], and death among children under five [14].

Despite ongoing efforts to apply different preventive measures at all levels, LBW and its complications are still prevalent in many countries [14,15,16,17], especially African countries [18,19,20,21,22,23,24,25,26]. Moreover, several factors, such as being a young/adolescent mother [27], a maternal age of >34 years, unplanned pregnancy, family size, alcohol abuse [28], maternal factors (e.g., education, undernutrition [29,30], and a low level of antenatal care (ANC)) [31], late antenatal attendance [15], birth interval, maternal hypertension [32], maternal anthropometric measurements [33], maternal smoking [34], household second-hand smoke, socioeconomic status [35], being unmarried [36], inadequate gestational weight gain [37], maternal anemia [38], maternal infection [39], and female sex [40], are reportedly associated with LBW.

Sudan is the third-largest country in Africa. Several studies have reported a high prevalence of LBW in different regions of Sudan [23,41,42,43,44,45,46]. Moreover, it has been reported that LBW is the leading cause of neonatal/perinatal mortality in Sudan [47]. There is a need to investigate the prevalence of LBW, along with the factors associated with it, to generate data that can help health planners reduce its incidence. This task is a public health priority worldwide, particularly in resource-limited countries such as Sudan. Decreasing the incidence of LBW is part of the UN’s Sustainable Development Goal (SDG) 3.2, which targets reducing neonatal mortality to below 12 per 1000 live births by 2030 [48]. Because no systematic reviews or meta-analyses have addressed LBW in Sudan, we conducted this systematic review and meta-analysis to estimate the pooled prevalence of LBW and its associated factors.

## 2. Materials and Methods

### 2.1. Data Extraction

The Preferred Reporting Items for Systematic Reviews and Meta-Analyses (PRISMA) guidelines were followed in this systematic review and meta-analysis [49]. The Joanna Briggs Institute Meta-Analysis of Statistics Assessment and Review Instrument was used to assess the included studies [50].

The following search strategy, prepared according to the PICOS protocol, was used:

P (population): neonates OR newborns

I (intervention/exposure): birth weight OR LBW OR infant

C (comparison): average birth weight OR without LBW

O (outcome): prevalence OR associated factors OR predictors

S (study design): cohort OR cross-sectional

A search strategy combining free-text terms, title and abstract words, and medical subject headings for each exposure, participant, and study design was employed. These searches were then combined using Boolean operators. Additional eligible studies on LBW were sought by reviewing the reference lists of the identified articles. The major databases—PUBMED/MEDLINE, Cochrane Library, Google, Google Scholar, and African Index Medicus—were used to identify published articles. The reference lists of the identified studies were checked to retrieve additional articles. The key terms used in the PubMed search were as follows: [(newborn [MeSH Terms] OR neonate OR child OR infant OR children AND (birth weight [MeSH Terms] OR small for gestation OR low birth weight OR underweight OR normal birth weight OR abnormal birth weight) AND (Sudan)].

### 2.2. Inclusion Criteria

#### 2.2.1. Study Scope

All studies that reported the prevalence of LBW in Sudan and associated factors were included in this systematic review and meta-analysis.

#### 2.2.2. Study Design

Cross-sectional or cohort studies were included.

#### 2.2.3. Language

We included English-language articles reporting the prevalence of LBW or at least one associated factor.

#### 2.2.4. Publication and Publication Year

Articles published before January 2025 were included.

### 2.3. Exclusion Criteria

Articles published in languages other than English, case reports, review articles, studies that did not report specific data on LBW, studies assessing LBW under particular conditions, such as malaria and preeclampsia, editorials, and qualitative studies were excluded from the analysis.

Outcome measurement

We aimed to have two findings/measurements. The primary outcome was the prevalence of LBW, and the secondary outcome constituted the factors (socioeconomic, maternal, and neonatal) associated with LBW.

Operation definition

LBW was defined as a birth weight of less than 2500 g [1].

### 2.4. Quality of the Studies

The Modified Newcastle–Ottawa scale (NOS) was used to assess the quality of the included studies [51]. It uses a star system (with a maximum of 9 stars) to indicate study quality across three major domains: participant selection, comparability of study groups, and ascertainment of outcomes of interest. Studies with a score of ≥7 stars were considered high-quality (Table 1).

Two investigators (EMM and AA) independently identified studies eligible for this meta-analysis. Any disagreements were resolved through discussion with the other researcher (IA). For each included analysis, the following data were input into an Excel sheet: name(s) of author(s), year of publication, region of study, neonate sample size, and number of neonates with LBW (see Supplementary File S1).

### 2.5. Data Analysis and Heterogeneity Assessment

The “metacont” function in the meta package [59] of the open-source statistical software R version 4.0.3 (The R Foundation for Statistical Computing, Vienna, Austria) was used to perform meta-analyses of the pooled prevalence of LBW. The heterogeneity of the included studies was evaluated using the Cochrane Q statistic and the I^2^ statistic. A Cochrane Q with *p* < 0.10 and I^2^ > 50 was employed as a standard to indicate the heterogeneity of the included studies [60]. According to the Cochrane Q and I^2^ analyses, a random-effects or fixed-effects model was used to combine the included studies. In the meta-analysis, we did not observe any significant heterogeneity. Therefore, the corresponding tests (sensitivity analysis, subgroup analysis, and meta-regression) were not performed. Publication bias was assessed using Egger’s and Begg’s tests.

## 3. Results

**Figure 1:**
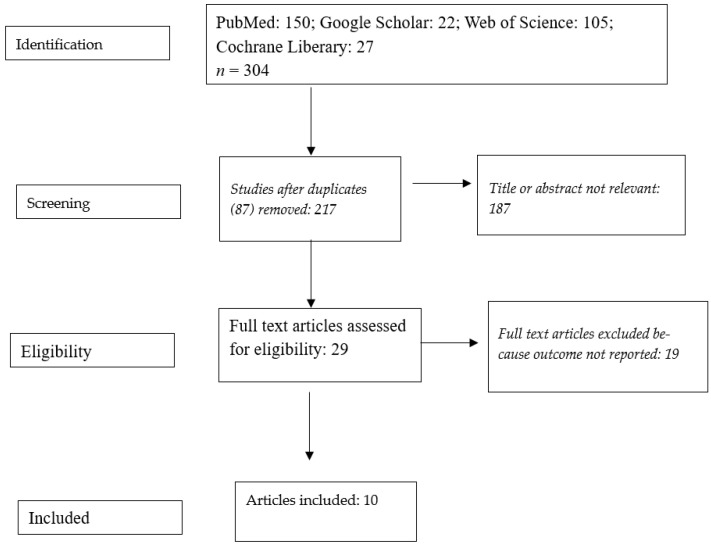
Flow chart showing how studies on low birth weight in Sudan were identified and selected.

After excluding articles for various reasons (e.g., titles and abstracts were not relevant, the outcome measure was not identified, LBW was assessed in regard to a specific disease (e.g., preeclampsia)), we used 10 articles to determine the prevalence and associated factors [41,45,46,52,53,54,55,56,57,58] (Figure 1). Sample sizes ranged from 253 neonates in Rabak [56] to 2818 neonates in Khartoum [52].

**Figure 2:**
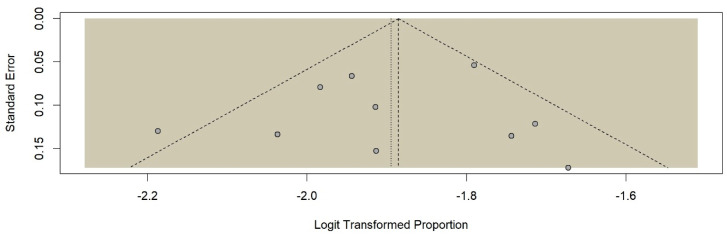
Funnel plot of the publication bias of the studies on low birth weight in Sudan.

**Figure 3:**
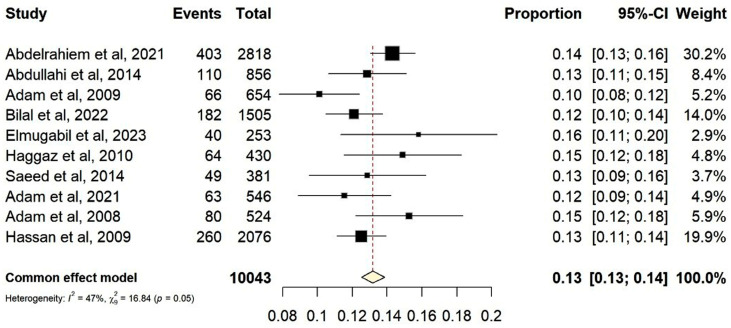
Forest plot of the pooled prevalence of low birth weight in Sudan [41,45,46,52,53,54,55,56,57,58].

All included articles were high-quality cross-sectional studies that used face-to-face questionnaires (Table 1). All studies were hospital-based. Five (50.0%) of these studies were conducted in Khartoum [41,53,55,57,58]. A funnel plot showing a symmetrical distribution is given in Figure 2. Egger’s regression test yielded a *p*-value of 0.063, which indicates the absence of publication bias. The prevalence of LBW ranged from 10.0% in Khartoum [55] to 15.8% in Rabak [56]. The overall pooled prevalence of LBW was 13.0% (95% CI [13.0, 14.0]; I^2^ = 47.0%, *p* = 0.05), without significant heterogeneity (Figure 3).

### Factors Associated with Low Birth Weight

Maternal undernutrition was the most commonly reported factor associated with LBW across four studies [41,46,53,54]. Gestational age was inversely associated with LBW, as reported in three [41,56,58]. Moreover, three studies reported associations between low-level ANC attendance and LBW [41,44,53] (Table 2 and Table 3).

Three studies reported an inverse association between gestational age and LBW. Primiparity (AOR 1.4, 95% CI [1.1, 2.1]) [55], a short interpregnancy interval [55], not using iron–folic acid supplements (AOR = 3.33, CI [1.47, −5.88]) [53], low maternal BMI (AOR = 1.8, 95% CI [1.0, 3.2]) [46], low level of education (of the mother) [54], low level of perinatal/antenatal care (<2 visits) (AOR = 2.10, 95% CI [1.30, 3.57]), maternal undernutrition (AOR = 1.66, 95% CI [1.09, 2.53]) [41], and decreasing duration/gestational age (AOR = 0.80, 95% CI [0.66, 0.96]) [56] were associated with LBW. In Medani (AOR = 9.0, 95% CI [3.4, 23.8]) [44], White Nile (AOR = 4.70, 95% CI [2.06, 10.94]) [56], and Darfur (AOR: 5.1, 95% CI [1.7, 15.2]) [45], anemia was associated with LBW (Table 2 and Table 3). In all these studies, the information was obtained from participants rather than from records. Moreover, with the exception of one study [54], in nine studies the associated factors were adjusted for confounders (Table 2).

## 4. Discussion

The pooled prevalence of LBW in this systematic review–meta-analysis was 13.0%. This is similar to the prevalence of LBW reported in Addis Ababa, Ethiopia (13.1%) [20], and Ghana (13.5%) [24], as well as in an extensive survey of 9734 births in Jordan (13.8%) [14]. The pooled prevalence of LBW in this systematic review was higher than in a similar study conducted in South Sudan (11.4%) [26]. Moreover, the detected pooled prevalence of LBW in our study was significantly higher than the recently reported value of 5.7% among 33,585 enrolled newborns in the Demographic and Health Survey (DHS) of Saharan African countries [31]. Likewise, the pooled prevalence of LBW in our analysis was higher than that (8.5%) reported in a meta-analysis that included 14 articles and 93,924 neonates in Iran [16]. However, the pooled prevalence in our systematic review and meta-analysis is lower than that (17.3%) reported in a meta-analysis of 30 studies and 55,085 neonates in Ethiopia [61]. Likewise, our pooled prevalence of LBW is lower than the reported prevalence (17.29%) for 175,240 neonates included in the fifth National Family Health Survey in India [17].

Differences in LBW prevalence across studies may be attributable to regional characteristics and factors associated with LBW. It has been reported that the prevalence of LBW is not evenly distributed across a country and that risk factors for LBW differ between countries and within regions of the same country [62]. Perhaps different factors associated with LBW, such as HIV [63] and malaria [64], have different prevalences across regions, and the prevalence of LBW varies across populations.

This systematic review and meta-analysis showed that primiparity, a short interpregnancy interval, lack of iron–folic acid supplementation, low maternal BMI, low education level (of the mother), low antenatal care level, maternal preterm birth, and maternal anemia were associated with LBW. This finding aligns with the results of a recent meta-analysis that included 30 studies with 55,085 participants in Ethiopia. Endalamaw et al. reported that maternal age, a short interpregnancy interval, a low BMI, and preterm birth were associated with LBW [61]. Likewise, in Jordan, preterm birth, maternal age, a low level of education (of the mother), and short birth interval were associated with LBW [14]. A previous meta-analysis showed that women who had fewer than four ANC visits were at a higher risk of delivering LBW neonates [31]. Gautam et al. showed that each increase in the number of ANC visits can lead to a 22 g increase in newborn birth weight as well as a 1.2 percentage reduction in the risk of delivering a LBW newborn [65]. It was recently shown that not only ANC or iron–folic acid supplementation but also multiple-micronutrient and calcium supplementation could prevent LBW relative to a placebo [66].

Three of the ten studies in this review reported an association between maternal anemia and LBW. In their meta-analysis, which included 68 articles, Figueiredo et al. reported that maternal anemia was associated with LBW (OR: 1.23, 95% CI [1.06, 1.43]) [67]. In Ethiopia, after adjusting for confounders (maternal age, parity, ANC), anemia was associated with LBW [32,68]. Anemia among pregnant women is one of the most significant health problems [69], including in Sudan [70]. Thus, preventive measures against anemia should be implemented across regions to reduce the prevalence of LBW. The difference between the results of this systematic review and meta-analysis and other results from other studies on different populations in Africa can be attributed to differences in the socioeconomic characteristics of the populations studied. For instance, while alcohol abuse [28], maternal smoking [34], and being unmarried are reportedly associated with LBW in African countries, smoking and alcohol consumption are rarely habits among women in Sudan. Moreover, according to the law, as well as tradition, women are not allowed to get pregnant without being married.

In summary, the main factors (maternal education, low AN levels, undernutrition, and anemia) associated with LBW are interrelated and interact. For example, it has been reported that low maternal education, not taking supplements, and not having nutritional education are associated with maternal anemia [32]. Moreover, it has been shown that, after controlling for the factors(maternal age, marital status, level of education, age of child, and wealth index), maternal anemia was associated with LBW [31].

### Strengths and Limitations

As for strengths, this study is the first systematic review and meta-analysis focused on Sudan that addresses a relevant public health topic with clear public health implications of great regional importance, thereby filling a genuine knowledge gap. It was elaborated in accordance with PRISMA guidelines, and the methodology is generally transparent. The databases searched are extensive (PubMed, Google Scholar, Cochrane, and African Index Medicus). Critically, a quality assessment was performed (Modified Newcastle–Ottawa Scale), and all studies that were included were rated as being of high quality. The sample size is reasonable, with 10 studies and more than 10,000 neonates included. Furthermore, the statistical analysis was appropriate.

However, this study has some weaknesses/limitations. Although the sample size is reasonable, it is small (*n* = 10), which limits the robustness of the conclusions. A major limitation of this review is its heavy reliance on cross-sectional studies, restricting the ability to establish causal relationships between risk factors and low birth weight. Consequently, the observed associations should be interpreted with caution, as they do not allow for definitive inferences regarding causality. The review was restricted to studies published in English, which may have introduced language bias and led to the exclusion of relevant non-English research. Risk factors were summarized descriptively using reported adjusted odds ratios, without pooling. Some key variables, such as maternal diet, mental health, infections (malaria, HIV, etc.), and socioeconomic status after pursuing an education, are missing. Although the included studies were conducted across various regions of Sudan, some, such as Northern Sudan (near Egypt) and Eastern Sudan (along the Red Sea), lack published data on LBW.

## 5. Conclusions

This review reveals a high prevalence of LBW in Sudan. The factors identified may help health planners and policymakers design and implement interventions to prevent LBW. Thus, greater efforts are needed to increase ANC levels and ensure iron and folic acid supplementation. Preventive measures against maternal undernutrition and anemia (nutrition) should be implemented across regions to reduce the prevalence of LBW.

## Figures and Tables

**Table 1:** Modified Newcastle–Ottawa scale scores of the included studies on low birth weight in Sudan.

Authors	Year of Publication	Selection	Comparability	Outcome	Total Rating
Abdelrahiem et al. [52]	2021	****	**	**	8
Abdullahi et al. [53]	2014	****	**	**	8
Adam et al. [54]	2021	****	**	**	8
Adam et al. [55]	2009	****	**	**	8
Adam et al. [46]	2008	****	**	**	8
Bilal et al. [41]	2022	****	**	**	8
Elmugabil et al. [56]	2023	****	**	**	8
Haggaz et al. [45]	2010	****	**	**	8
Hassan et al. [57]	2009	****	**	**	8
Saeed et al. [58]	2014	****	**	**	8

**Table 2:** Characteristics of the studies, confounders, and factors associated with low birth weight in Sudan.

Authors	Regions	Sample Size	Associated Factors	Confounders
Abdullahi et al. [53]	Khartoum	2818	Not using iron–folic acid	Maternal age, occupation, residence, and newborn sex
Adam et al. [55]	Khartoum	654	Previous history of preterm birth and a short interpregnancy interval	Maternal age, parity, education, residence, ANC, and anemia
Adam et al. [46]	New Halfa	524	Maternal undernutrition	Maternal age, parity, anemia, malaria
Adam et al. [54]	Elobeid	546	Maternal education and undernutrition	Maternal age, occupation, residence, and newborn sex
Bilal et al. [41]	Khartoum	1505	Low level of antenatal care, maternal undernutrition, and gestational age	Maternal age, parity, education, occupation, residence, history of miscarriage, and newborn sex
Elmugabil et al. [56]	Rabak	253	Maternal anemia and gestational age	Maternal age, parity, education, occupation, residence, history of miscarriage, ANC, and newborn sex
Haggaz et al. [45]	Elfasher Darfur	430	Maternal anemia	Maternal age, parity, education, occupation, BMI, newborn sex
Hassan et al. [57]	Khartoum	2076	Primipara, low antenatal care, and maternal undernutrition	Maternal age, education, occupation, hemoglobin, and newborn sex
Saeed et al. [58]	Khartoum	381	Low maternal education, bleeding in early pregnancy, and gestational age	ANC, malaria, anemia
Elhassan [44]	Medani	97 (case-controls)	low level of antenatal care, anemia	Maternal age, parity, education, occupation, nutrition, and interpregnancy interval

**Table 3:** Factors associated with low birth weight in Sudan.

Factors Associated with Low Birth Weight	Authors and References			
Maternal undernutrition	Adam et al. [46]	Adam et al. [54]	Bilal et al. [41]	Hassan et al. [53]
Gestational age	Elmugabil et al. [56]	Saeed et al. [58]	Bilal et al. [41]	
Low antenatal care level	Bilal et al. [41]	Hassan et al. [53]	Elhassan [44]	
Maternal anemia	Haggaz et al. [45]	Elmugabil et al. [56]	Elhassan [44]	
Low level of maternal education	Adam et al. [54]	Saeed et al. [58]		
Primiparas	Hassan et al. [53]			
Bleeding in early pregnancy	Saeed et al. [58]			
short interpregnancy interval	Adam et al. [55]			
Not using iron–folic acid	Abdullahi et al. [53]			
Previous history of preterm birth	Adam et al. [55]			

## Data Availability

The datasets used and analyzed in this study are included in the paper.

## Supplementary Materials

The following supporting information can be downloaded at: https://www.mdpi.com/article/10.3390/children13020274/s1, File S1: The details of the included studies.

## Abbreviations

The following abbreviations are used in this manuscript:

ANC	antenatal care
AOR	adjusted odds ratio
CI	confidence interval
IQR	interquartile range
WHO	World Health Organization
